# 2-Chloro-*N*-{5-[(4*R*,5*R*,10*S*)-dehydro­abiet-4-yl]-1,3,4-thia­diazol-2-yl}benzamide

**DOI:** 10.1107/S1600536811007665

**Published:** 2011-03-09

**Authors:** Qijin Mo, Wengui Duan, Xianli Ma, Jianxin Huang, Zhen Ma

**Affiliations:** a College of Chemistry & Chemical Engineering, Guangxi University, Nanning 530004, People’s Republic of China; b College of Pharmacy, Guilin Medical University, Guilin 541004, People’s Republic of China

## Abstract

There are two independent mol­ecules in the asymmetric unit of the title compound, C_28_H_32_ClN_3_OS (systematic name: 2-chloro-*N*-{5-[(1*R*,4a*S*,10a*R*)-7-isopropyl-1,4a-dimethyl-1,2,3,4,4a,9,10,10a-octa­hydro­phenanthren-1-yl]-1,3,4-thia­diazol-2-yl}benzamide). In each mol­ecule, the cyclo­hexyl ring attached to the thia­diazole fragment adopts a classic chair conformation with two of its two methyl groups in the axial positions. In the crystal, pairs of inter­molecular N—H⋯N hydrogen bonds link the mol­ecules into centrosymmetric dimers, which are further linked *via* C—H⋯π inter­actions.

## Related literature

For background to the uses of rosin, see: Song (2004 ▶). For the isolation of dehydro­abietic acid, the major component of disproportionated rosin, see: Xu *et al.* (2009 ▶). For the biolog­ical activity of dehydro­abietic derivatives, see: Sepulveda *et al.* (2005 ▶). For the synthesis of the title compound, see: Liu *et al.* (2009 ▶). For related structures, see: Rao *et al.* (2007 ▶); Gu & Wang (2009 ▶). For standard bond lengths, see: Allen *et al.* (1987 ▶).
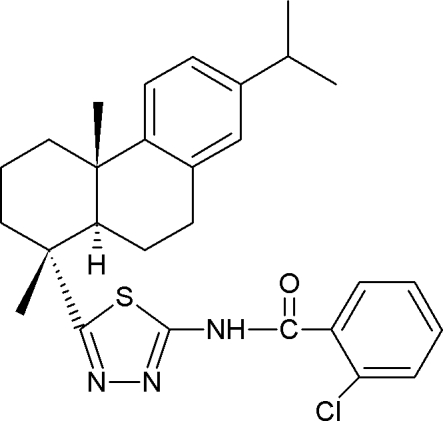


## Experimental

### 

#### Crystal data

C_28_H_32_ClN_3_OS
*M* *_r_* = 494.08
Monoclinic, 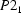

*a* = 7.9707 (16) Å
*b* = 31.874 (6) Å
*c* = 10.863 (2) Å
β = 107.24 (3)°
*V* = 2635.7 (9) Å^3^
*Z* = 4
Mo *K*α radiation
μ = 0.25 mm^−1^
*T* = 293 K
0.43 × 0.38 × 0.32 mm

#### Data collection

Bruker SMART CCD area-detector diffractometer
Absorption correction: multi-scan (*SADABS*; Sheldrick, 1996 ▶) *T*_min_ = 0.898, *T*_max_ = 0.923
16616 measured reflections
10596 independent reflections
6952 reflections with *I* > 2σ(*I*)
*R*_int_ = 0.032

#### Refinement

*R*[*F* ^2^ > 2σ(*F* ^2^)] = 0.045
*wR*(*F* ^2^) = 0.105
*S* = 1.01
10596 reflections
613 parameters
1 restraint
H-atom parameters constrained
Δρ_max_ = 0.46 e Å^−3^
Δρ_min_ = −0.23 e Å^−3^
Absolute structure: Flack (1983 ▶), 4741 Friedel pairs
Flack parameter: 0.03 (5)

### 

Data collection: *SMART* (Bruker, 2001 ▶); cell refinement: *SAINT* (Bruker, 2002 ▶); data reduction: *SAINT*; program(s) used to solve structure: *SHELXS97* (Sheldrick, 2008 ▶); program(s) used to refine structure: *SHELXS97* (Sheldrick, 2008 ▶); molecular graphics: *SHELXTL* (Sheldrick, 2008 ▶); software used to prepare material for publication: *SHELXTL*.

## Supplementary Material

Crystal structure: contains datablocks I, global. DOI: 10.1107/S1600536811007665/lx2187sup1.cif

Structure factors: contains datablocks I. DOI: 10.1107/S1600536811007665/lx2187Isup2.hkl

Additional supplementary materials:  crystallographic information; 3D view; checkCIF report

## Figures and Tables

**Table 1:** Hydrogen-bond geometry (Å, °) *Cg* is the centroid of the C23–C28 2-chloro­phenyl ring.

*D*—H⋯*A*	*D*—H	H⋯*A*	*D*⋯*A*	*D*—H⋯*A*
N3—H3*A*⋯N5	0.86	2.03	2.882 (3)	172
N6—H6*A*⋯N2	0.86	2.14	2.982 (3)	166
C42—H42*B*⋯*Cg*^i^	0.97	2.65	3.462 (3)	141

Symmetry code: (i) 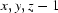.

**Fig. 1.**
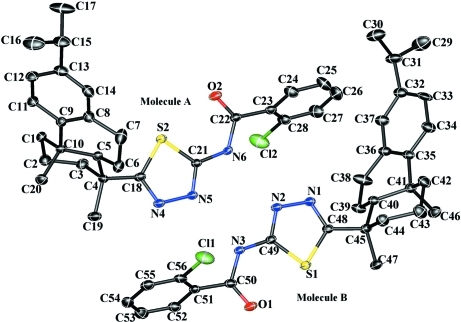
The molecular structure of the title compound with the atom numbering scheme. Displacement ellipsoids are drawn at the 30% probability level. H atoms are presented as a small spheres of arbitrary radius.

**Fig. 2.**
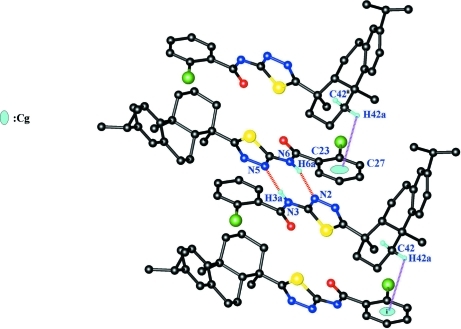
A view of the N—H···N and C—H···π interactions (dotted lines) in the crystal structure of the title compound. [Symmetry codes: (i) x, y, -1 + z; (ii) x, y, 1 + z.]

## supplementary crystallographic information

### Comment

Rosin, known as an important chemical raw material, is widely used in papermaking, adhesives, paint, printing ink, rubber, food, and other industries (Song, 2004). Dehydroabietic acid is the dominant component of disproportionated rosin produced by catalytic disproportionation of rosin. Therefore, the search for novel bioactive compounds and the study of their pharmacological properties constitute a matter of current interest. Hence, a series of dehydroabietic acid derivatives bearing heterocyclic ring were synthesized and their properties in many fileds have been researched. Herein, we report the crystal structure of the title compound.

The asymmetric unit of the title compound is shown in Fig. 1. There are two independent molecules [A and B] and all bond lengths and angles are within normal ranges (Allen *et al.*, 1987). In each molecule, the cyclohexyl ring having the thiadiazole fragment adopts a classic chair conformation with two methyl groups in the axial positions. The crystal packing (Fig. 2) is stabilized by intermolecular N—H···N hydrogen bonds between the hydrogen of the amide group and the thiadiazole N atom (see; Table 1). The crystal packing (Fig. 2) is further stabilized by intermolecular C—H···π interactions between a cyclohexyl H atom and the 2-chlorophenyl ring (Table 1; *Cg* is the centroid of the C23–C28 2-chlorophenyl ring).

### Experimental

2-Amino-5-dehydroabietyl-1,3,4-thiadiazole was synthesized from dehydroabietic acid at first. Then a solution of the *o*-chlorobenzoyl chloride (8 mmol) in methylene chloride (5 ml) was added dropwise to a mixture of 2-amino-5-dehydroabietyl-1,3,4-thiadiazole (2.84 g, 8 mmol), triethylamine (1.16 ml, 10 mmol) and methylene chloride (15 ml) with constant stirring at 0–5°C. The reaction mixture was stirred for 45 min at this temperature, then for 5 h at room temperature, followed by evaporation of the mixture *in vacuo* to afford a yellowish solid, which was washed with water and recrystalized to give the title compound, 2-(2-chlorobenzamido)-5-[(4R,5R,10S)dehydroabietyl]-1,3,4-thiadiazole. Single crystals of the title compound suitable for an X-ray diffraction study were obtained by slow evaporation of an acetone solution at room temperature over a period of 10 d.

### Refinement

All H atoms were positioned geometrically and refined using a riding model with C—H = 0.93–0.98 Å and N—H=0.86 Å with *U*_iso_(H) = 1.5Ueq(C) for methyl H atoms and *Uiso*(H) = 1.2*Ueq*(C) for all other H atoms.

### Crystal data

**Table:**

C_28_H_32_ClN_3_OS	*F*(000) = 1048
*M**_r_* = 494.08	*D*_x_ = 1.245 Mg m^−^^3^
Monoclinic, *P*2_1_	Mo *K*α radiation, λ = 0.71073 Å
Hall symbol: P 2yb	Cell parameters from 10596 reflections
*a* = 7.9707 (16) Å	θ = 2.0–27.0°
*b* = 31.874 (6) Å	µ = 0.25 mm^−^^1^
*c* = 10.863 (2) Å	*T* = 293 K
β = 107.24 (3)°	Block, colorless
*V* = 2635.7 (9) Å^3^	0.43 × 0.38 × 0.32 mm
*Z* = 4	

### Data collection

**Table:**

Bruker SMART CCD area-detector diffractometer	10596 independent reflections
Radiation source: fine-focus sealed tube	6952 reflections with *I* > 2σ(*I*)
graphite	*R*_int_ = 0.032
Detector resolution: 0 pixels mm^-1^	θ_max_ = 27.0°, θ_min_ = 2.0°
φ and ω scans	*h* = −10→9
Absorption correction: multi-scan (*SADABS*; Sheldrick, 1996)	*k* = −40→37
*T*_min_ = 0.898, *T*_max_ = 0.923	*l* = −13→13
16616 measured reflections	

### Refinement

**Table:**

Refinement on *F*^2^	Secondary atom site location: difference Fourier map
Least-squares matrix: full	Hydrogen site location: inferred from neighbouring sites
*R*[*F*^2^ > 2σ(*F*^2^)] = 0.045	H-atom parameters constrained
*wR*(*F*^2^) = 0.105	*w* = 1/[σ^2^(*F*_o_^2^) + (0.0398*P*)^2^] where *P* = (*F*_o_^2^ + 2*F*_c_^2^)/3
*S* = 1.01	(Δ/σ)_max_ = 0.002
10596 reflections	Δρ_max_ = 0.46 e Å^−^^3^
613 parameters	Δρ_min_ = −0.23 e Å^−^^3^
1 restraint	Absolute structure: Flack (1983), 4741 Friedel pairs
Primary atom site location: structure-invariant direct methods	Flack parameter: 0.03 (5)

### Special details

**Table:**

Geometry. All e.s.d.'s (except the e.s.d. in the dihedral angle between two l.s. planes) are estimated using the full covariance matrix. The cell e.s.d.'s are taken into account individually in the estimation of e.s.d.'s in distances, angles and torsion angles; correlations between e.s.d.'s in cell parameters are only used when they are defined by crystal symmetry. An approximate (isotropic) treatment of cell e.s.d.'s is used for estimating e.s.d.'s involving l.s. planes.
Refinement. Refinement of *F*^2^ against ALL reflections. The weighted *R*-factor *wR* and goodness of fit *S* are based on *F*^2^, conventional *R*-factors *R* are based on *F*, with *F* set to zero for negative *F*^2^. The threshold expression of *F*^2^ > σ(*F*^2^) is used only for calculating *R*-factors(gt) *etc*. and is not relevant to the choice of reflections for refinement. *R*-factors based on *F*^2^ are statistically about twice as large as those based on *F*, and *R*- factors based on ALL data will be even larger.

### Fractional atomic coordinates and isotropic or equivalent isotropic displacement parameters (Å^2^)

**Table:**

	*x*	*y*	*z*	*U*_iso_*/*U*_eq_	
S1	0.52338 (11)	0.65114 (3)	1.44011 (7)	0.0497 (2)	
S2	0.28189 (11)	0.72225 (3)	0.73498 (7)	0.0514 (2)	
Cl1	0.30129 (14)	0.79642 (3)	1.20908 (12)	0.0902 (3)	
Cl2	0.49960 (14)	0.57382 (3)	0.94336 (13)	0.0968 (4)	
O1	0.2265 (3)	0.70066 (9)	1.4129 (2)	0.0801 (8)	
O2	0.5858 (3)	0.67606 (8)	0.7564 (2)	0.0719 (7)	
N1	0.6791 (3)	0.62791 (8)	1.2769 (2)	0.0554 (7)	
N2	0.5440 (3)	0.65378 (8)	1.2088 (2)	0.0538 (7)	
N3	0.3140 (3)	0.69429 (8)	1.2328 (2)	0.0454 (6)	
H3A	0.2929	0.7018	1.1536	0.054*	
N4	0.1235 (3)	0.73933 (8)	0.9030 (2)	0.0533 (7)	
N5	0.2624 (3)	0.71246 (8)	0.9642 (2)	0.0499 (7)	
N6	0.4935 (3)	0.67391 (8)	0.9321 (2)	0.0445 (6)	
H6A	0.5125	0.6636	1.0082	0.053*	
C29	1.2728 (7)	0.40459 (13)	1.0712 (4)	0.1108 (17)	
H29A	1.2768	0.3937	0.9898	0.166*	
H29B	1.2288	0.3834	1.1164	0.166*	
H29C	1.3888	0.4127	1.1216	0.166*	
C30	1.1898 (6)	0.47207 (13)	0.9565 (4)	0.0929 (13)	
H30A	1.2037	0.4578	0.8824	0.139*	
H30B	1.2944	0.4878	0.9972	0.139*	
H30C	1.0915	0.4909	0.9302	0.139*	
C31	1.1585 (6)	0.44060 (13)	1.0499 (4)	0.0782 (11)	
H31A	1.0418	0.4292	1.0062	0.094*	
C32	1.1362 (5)	0.46210 (10)	1.1685 (3)	0.0595 (9)	
C33	1.2762 (5)	0.48006 (13)	1.2603 (4)	0.0748 (11)	
H33A	1.3891	0.4764	1.2540	0.090*	
C34	1.2526 (5)	0.50371 (12)	1.3632 (4)	0.0690 (10)	
H34A	1.3501	0.5157	1.4223	0.083*	
C35	1.0877 (4)	0.50968 (9)	1.3787 (3)	0.0477 (8)	
C36	0.9465 (4)	0.48940 (10)	1.2889 (3)	0.0496 (8)	
C37	0.9756 (5)	0.46662 (10)	1.1890 (3)	0.0563 (9)	
H37A	0.8797	0.4534	1.1318	0.068*	
C38	0.7639 (5)	0.49269 (11)	1.2989 (4)	0.0734 (11)	
H38A	0.7358	0.4665	1.3336	0.088*	
H38B	0.6840	0.4956	1.2125	0.088*	
C39	0.7276 (4)	0.52832 (10)	1.3808 (3)	0.0555 (9)	
H39A	0.7067	0.5166	1.4574	0.067*	
H39B	0.6216	0.5428	1.3324	0.067*	
C40	0.8789 (4)	0.56011 (8)	1.4217 (3)	0.0425 (7)	
H40A	0.8901	0.5716	1.3408	0.051*	
C41	1.0566 (4)	0.53761 (9)	1.4854 (3)	0.0480 (7)	
C42	1.1993 (4)	0.57089 (11)	1.5292 (4)	0.0641 (9)	
H42A	1.3076	0.5572	1.5773	0.077*	
H42B	1.2189	0.5836	1.4535	0.077*	
C43	1.1580 (5)	0.60547 (12)	1.6125 (4)	0.0763 (11)	
H43A	1.2547	0.6253	1.6365	0.092*	
H43B	1.1438	0.5934	1.6908	0.092*	
C44	0.9881 (4)	0.62848 (10)	1.5379 (3)	0.0608 (9)	
H44A	0.9619	0.6500	1.5926	0.073*	
H44B	1.0062	0.6422	1.4631	0.073*	
C45	0.8311 (4)	0.59840 (9)	1.4937 (3)	0.0484 (8)	
C46	1.0591 (5)	0.50884 (11)	1.6010 (3)	0.0679 (10)	
H46A	1.1732	0.4963	1.6342	0.102*	
H46B	0.9724	0.4872	1.5730	0.102*	
H46C	1.0333	0.5252	1.6674	0.102*	
C47	0.7623 (5)	0.58685 (11)	1.6091 (3)	0.0626 (9)	
H47A	0.7361	0.6121	1.6482	0.094*	
H47B	0.8506	0.5712	1.6716	0.094*	
H47C	0.6579	0.5702	1.5790	0.094*	
C48	0.6863 (4)	0.62389 (9)	1.3968 (3)	0.0418 (7)	
C49	0.4519 (4)	0.66797 (9)	1.2826 (3)	0.0426 (7)	
C50	0.2062 (4)	0.70969 (10)	1.3003 (3)	0.0497 (8)	
C51	0.0620 (4)	0.73779 (9)	1.2263 (3)	0.0446 (7)	
C52	−0.1106 (4)	0.72410 (11)	1.2042 (3)	0.0549 (8)	
H52A	−0.1326	0.6981	1.2351	0.066*	
C53	−0.2474 (5)	0.74882 (12)	1.1371 (3)	0.0660 (10)	
H53A	−0.3620	0.7392	1.1217	0.079*	
C54	−0.2178 (5)	0.78801 (12)	1.0918 (3)	0.0645 (10)	
H54A	−0.3117	0.8046	1.0463	0.077*	
C55	−0.0479 (5)	0.80212 (12)	1.1148 (3)	0.0643 (10)	
H55A	−0.0264	0.8285	1.0864	0.077*	
C56	0.0897 (4)	0.77689 (10)	1.1801 (3)	0.0516 (8)	
C17	0.4778 (7)	0.90964 (14)	0.2173 (6)	0.1203 (19)	
H17A	0.5556	0.9249	0.1810	0.180*	
H17B	0.3730	0.9023	0.1502	0.180*	
H17C	0.5350	0.8845	0.2577	0.180*	
C15	0.4303 (5)	0.93651 (12)	0.3162 (4)	0.0800 (11)	
H15A	0.5392	0.9414	0.3858	0.096*	
C16	0.3622 (6)	0.97866 (13)	0.2633 (5)	0.1070 (16)	
H16A	0.4455	0.9919	0.2276	0.160*	
H16B	0.3450	0.9958	0.3312	0.160*	
H16C	0.2524	0.9753	0.1971	0.160*	
C13	0.3063 (5)	0.91287 (10)	0.3756 (4)	0.0602 (9)	
C14	0.3584 (4)	0.89840 (10)	0.4994 (4)	0.0639 (9)	
H14A	0.4713	0.9048	0.5508	0.077*	
C8	0.2500 (4)	0.87438 (10)	0.5529 (3)	0.0540 (8)	
C9	0.0814 (4)	0.86399 (9)	0.4786 (3)	0.0444 (7)	
C11	0.0256 (4)	0.88025 (10)	0.3529 (3)	0.0560 (8)	
H11A	−0.0889	0.8749	0.3027	0.067*	
C12	0.1319 (5)	0.90370 (11)	0.3003 (3)	0.0626 (9)	
H12A	0.0898	0.9135	0.2161	0.075*	
C7	0.3267 (5)	0.85860 (14)	0.6897 (4)	0.0857 (12)	
H7A	0.4205	0.8390	0.6916	0.103*	
H7B	0.3783	0.8821	0.7445	0.103*	
C6	0.1969 (4)	0.83739 (11)	0.7452 (3)	0.0603 (9)	
H6B	0.1296	0.8584	0.7749	0.072*	
H6C	0.2589	0.8203	0.8184	0.072*	
C5	0.0729 (4)	0.80979 (9)	0.6425 (3)	0.0412 (7)	
H5B	0.1503	0.7945	0.6030	0.049*	
C10	−0.0424 (4)	0.83779 (9)	0.5317 (3)	0.0404 (7)	
C1	−0.1557 (4)	0.80806 (9)	0.4271 (3)	0.0472 (7)	
H1B	−0.2385	0.8248	0.3622	0.057*	
H1C	−0.0797	0.7938	0.3854	0.057*	
C2	−0.2580 (4)	0.77503 (10)	0.4787 (3)	0.0561 (8)	
H2B	−0.3415	0.7889	0.5144	0.067*	
H2C	−0.3231	0.7571	0.4086	0.067*	
C3	−0.1317 (4)	0.74846 (10)	0.5827 (3)	0.0519 (8)	
H3B	−0.0531	0.7335	0.5450	0.062*	
H3C	−0.1983	0.7279	0.6145	0.062*	
C4	−0.0228 (4)	0.77502 (10)	0.6959 (3)	0.0448 (7)	
C20	−0.1634 (4)	0.86973 (11)	0.5727 (3)	0.0623 (10)	
H20A	−0.2291	0.8855	0.4987	0.093*	
H20B	−0.0929	0.8885	0.6362	0.093*	
H20C	−0.2430	0.8549	0.6083	0.093*	
C19	−0.1391 (5)	0.79158 (11)	0.7772 (3)	0.0663 (10)	
H19A	−0.1936	0.7683	0.8067	0.099*	
H19B	−0.2282	0.8098	0.7253	0.099*	
H19C	−0.0676	0.8069	0.8501	0.099*	
C18	0.1151 (4)	0.74680 (9)	0.7841 (3)	0.0443 (7)	
C21	0.3522 (4)	0.70156 (8)	0.8882 (3)	0.0394 (7)	
C22	0.6025 (4)	0.66216 (9)	0.8636 (3)	0.0447 (7)	
C28	0.7144 (4)	0.59235 (10)	0.9710 (3)	0.0569 (9)	
C23	0.7442 (4)	0.63191 (9)	0.9305 (3)	0.0446 (7)	
C27	0.8494 (6)	0.56572 (12)	1.0292 (4)	0.0773 (11)	
H27A	0.8259	0.5392	1.0555	0.093*	
C26	1.0188 (6)	0.57842 (15)	1.0484 (4)	0.0890 (14)	
H26A	1.1110	0.5604	1.0876	0.107*	
C25	1.0531 (5)	0.61742 (14)	1.0102 (4)	0.0811 (12)	
H25A	1.1685	0.6262	1.0246	0.097*	
C24	0.9155 (5)	0.64378 (11)	0.9499 (3)	0.0653 (10)	
H24A	0.9396	0.6701	0.9221	0.078*	

### Atomic displacement parameters (Å^2^)

**Table:**

	*U*^11^	*U*^22^	*U*^33^	*U*^12^	*U*^13^	*U*^23^
S1	0.0596 (5)	0.0572 (5)	0.0345 (4)	0.0170 (4)	0.0173 (4)	0.0088 (4)
S2	0.0595 (5)	0.0619 (5)	0.0350 (4)	0.0233 (4)	0.0174 (4)	0.0106 (4)
Cl1	0.0772 (7)	0.0690 (7)	0.1314 (9)	−0.0054 (5)	0.0419 (7)	0.0057 (6)
Cl2	0.0791 (7)	0.0562 (6)	0.1599 (12)	−0.0030 (5)	0.0426 (7)	0.0089 (6)
O1	0.0897 (19)	0.113 (2)	0.0447 (15)	0.0469 (16)	0.0317 (14)	0.0243 (14)
O2	0.0875 (18)	0.0914 (17)	0.0450 (14)	0.0372 (14)	0.0325 (14)	0.0237 (13)
N1	0.0585 (17)	0.0676 (18)	0.0421 (16)	0.0281 (15)	0.0181 (13)	0.0142 (14)
N2	0.0621 (17)	0.0638 (17)	0.0402 (15)	0.0278 (14)	0.0223 (14)	0.0149 (14)
N3	0.0517 (16)	0.0531 (16)	0.0338 (14)	0.0150 (12)	0.0166 (13)	0.0094 (11)
N4	0.0616 (17)	0.0605 (17)	0.0418 (16)	0.0258 (14)	0.0215 (14)	0.0123 (13)
N5	0.0561 (17)	0.0588 (17)	0.0375 (14)	0.0246 (13)	0.0180 (13)	0.0128 (13)
N6	0.0538 (16)	0.0513 (15)	0.0280 (13)	0.0175 (12)	0.0115 (12)	0.0094 (11)
C29	0.177 (5)	0.070 (3)	0.090 (3)	0.041 (3)	0.045 (3)	0.005 (2)
C30	0.137 (4)	0.076 (3)	0.082 (3)	0.015 (3)	0.057 (3)	0.007 (2)
C31	0.100 (3)	0.078 (3)	0.072 (3)	0.028 (2)	0.049 (2)	0.009 (2)
C32	0.065 (3)	0.056 (2)	0.064 (2)	0.0106 (18)	0.029 (2)	0.0133 (18)
C33	0.051 (2)	0.099 (3)	0.088 (3)	0.025 (2)	0.041 (2)	0.013 (2)
C34	0.049 (2)	0.082 (3)	0.076 (3)	0.0087 (18)	0.017 (2)	0.001 (2)
C35	0.049 (2)	0.0442 (18)	0.053 (2)	0.0119 (15)	0.0198 (17)	0.0141 (15)
C36	0.054 (2)	0.0452 (18)	0.057 (2)	0.0072 (15)	0.0269 (18)	0.0058 (15)
C37	0.062 (2)	0.0461 (19)	0.062 (2)	0.0063 (15)	0.0204 (18)	0.0011 (16)
C38	0.062 (2)	0.062 (2)	0.101 (3)	−0.0085 (18)	0.033 (2)	−0.019 (2)
C39	0.049 (2)	0.0493 (19)	0.074 (2)	0.0058 (15)	0.0270 (18)	−0.0014 (16)
C40	0.0431 (18)	0.0412 (17)	0.0438 (18)	0.0064 (13)	0.0139 (14)	0.0070 (13)
C41	0.0495 (19)	0.0480 (18)	0.0452 (18)	0.0065 (14)	0.0118 (15)	0.0069 (14)
C42	0.0412 (19)	0.072 (2)	0.072 (2)	0.0059 (17)	0.0060 (17)	0.0001 (19)
C43	0.061 (2)	0.069 (2)	0.083 (3)	0.0004 (19)	−0.005 (2)	−0.010 (2)
C44	0.067 (2)	0.0479 (19)	0.058 (2)	0.0054 (17)	0.0027 (18)	−0.0058 (17)
C45	0.064 (2)	0.0423 (18)	0.0383 (18)	0.0084 (15)	0.0142 (16)	0.0027 (14)
C46	0.086 (3)	0.061 (2)	0.056 (2)	0.0218 (19)	0.021 (2)	0.0188 (18)
C47	0.078 (2)	0.068 (2)	0.0438 (19)	0.0313 (18)	0.0213 (18)	0.0168 (17)
C48	0.0447 (18)	0.0412 (17)	0.0399 (17)	0.0047 (14)	0.0130 (14)	0.0063 (14)
C49	0.052 (2)	0.0425 (17)	0.0339 (17)	0.0052 (14)	0.0128 (15)	0.0059 (13)
C50	0.051 (2)	0.057 (2)	0.045 (2)	0.0073 (15)	0.0192 (17)	0.0017 (16)
C51	0.048 (2)	0.050 (2)	0.0374 (17)	0.0132 (15)	0.0156 (15)	0.0020 (14)
C52	0.056 (2)	0.056 (2)	0.053 (2)	0.0085 (17)	0.0169 (17)	−0.0011 (17)
C53	0.060 (2)	0.070 (3)	0.059 (2)	0.0040 (19)	0.0048 (19)	−0.020 (2)
C54	0.067 (3)	0.072 (3)	0.049 (2)	0.028 (2)	0.0085 (18)	−0.0055 (18)
C55	0.083 (3)	0.060 (2)	0.053 (2)	0.022 (2)	0.026 (2)	0.0051 (18)
C56	0.052 (2)	0.057 (2)	0.049 (2)	0.0078 (17)	0.0200 (17)	−0.0085 (17)
C17	0.130 (4)	0.085 (3)	0.192 (5)	0.003 (3)	0.120 (4)	0.007 (3)
C15	0.075 (3)	0.076 (3)	0.099 (3)	−0.004 (2)	0.042 (2)	0.017 (2)
C16	0.128 (4)	0.068 (3)	0.146 (4)	−0.004 (3)	0.073 (4)	0.023 (3)
C13	0.054 (2)	0.052 (2)	0.080 (3)	0.0047 (16)	0.027 (2)	0.0099 (18)
C14	0.044 (2)	0.057 (2)	0.079 (3)	0.0034 (16)	0.0022 (19)	0.0116 (19)
C8	0.050 (2)	0.0482 (19)	0.056 (2)	−0.0006 (15)	0.0043 (17)	0.0103 (16)
C9	0.0482 (19)	0.0411 (17)	0.0403 (18)	0.0116 (14)	0.0074 (15)	0.0049 (14)
C11	0.051 (2)	0.064 (2)	0.049 (2)	0.0037 (16)	0.0086 (16)	0.0159 (17)
C12	0.060 (2)	0.072 (2)	0.058 (2)	0.0040 (18)	0.0208 (19)	0.0189 (18)
C7	0.081 (3)	0.083 (3)	0.065 (3)	−0.019 (2)	−0.020 (2)	0.019 (2)
C6	0.067 (2)	0.059 (2)	0.042 (2)	0.0027 (17)	−0.0044 (17)	0.0042 (16)
C5	0.0468 (18)	0.0395 (16)	0.0339 (16)	0.0106 (13)	0.0066 (14)	0.0013 (13)
C10	0.0419 (17)	0.0442 (16)	0.0334 (17)	0.0108 (13)	0.0087 (14)	0.0020 (13)
C1	0.0466 (18)	0.0549 (19)	0.0348 (17)	0.0064 (15)	0.0038 (14)	0.0079 (14)
C2	0.050 (2)	0.062 (2)	0.051 (2)	−0.0032 (16)	0.0058 (16)	0.0103 (17)
C3	0.055 (2)	0.0507 (19)	0.048 (2)	−0.0046 (15)	0.0115 (16)	0.0088 (15)
C4	0.0394 (17)	0.0565 (19)	0.0388 (17)	0.0161 (14)	0.0120 (15)	0.0080 (15)
C20	0.067 (2)	0.065 (2)	0.054 (2)	0.0263 (18)	0.0161 (18)	0.0076 (17)
C19	0.075 (2)	0.075 (2)	0.057 (2)	0.029 (2)	0.0327 (19)	0.0153 (18)
C18	0.056 (2)	0.0406 (17)	0.0385 (18)	0.0100 (14)	0.0166 (16)	0.0013 (14)
C21	0.0450 (18)	0.0393 (16)	0.0339 (16)	0.0099 (13)	0.0117 (14)	0.0050 (13)
C22	0.0527 (19)	0.0465 (18)	0.0358 (18)	0.0077 (14)	0.0146 (16)	0.0023 (14)
C28	0.061 (2)	0.050 (2)	0.064 (2)	0.0150 (16)	0.0237 (18)	0.0098 (17)
C23	0.050 (2)	0.0479 (19)	0.0369 (17)	0.0102 (15)	0.0136 (15)	−0.0028 (14)
C27	0.085 (3)	0.053 (2)	0.093 (3)	0.024 (2)	0.026 (3)	0.022 (2)
C26	0.086 (3)	0.080 (3)	0.088 (3)	0.046 (3)	0.006 (3)	0.014 (2)
C25	0.046 (2)	0.078 (3)	0.109 (3)	0.017 (2)	0.007 (2)	−0.008 (3)
C24	0.059 (2)	0.055 (2)	0.080 (3)	0.0098 (18)	0.018 (2)	0.0024 (18)

### Geometric parameters (Å, °)

**Table:**

S1—C49	1.721 (3)	C51—C56	1.385 (4)
S1—C48	1.739 (3)	C51—C52	1.394 (4)
S2—C21	1.722 (3)	C52—C53	1.367 (5)
S2—C18	1.756 (3)	C52—H52A	0.9300
Cl1—C56	1.737 (3)	C53—C54	1.389 (5)
Cl2—C28	1.751 (4)	C53—H53A	0.9300
O1—C50	1.220 (4)	C54—C55	1.377 (5)
O2—C22	1.216 (3)	C54—H54A	0.9300
N1—C48	1.294 (3)	C55—C56	1.375 (5)
N1—N2	1.383 (3)	C55—H55A	0.9300
N2—C49	1.318 (3)	C17—C15	1.508 (6)
N3—C49	1.361 (4)	C17—H17A	0.9600
N3—C50	1.376 (4)	C17—H17B	0.9600
N3—H3A	0.8600	C17—H17C	0.9600
N4—C18	1.295 (3)	C15—C16	1.499 (6)
N4—N5	1.402 (3)	C15—C13	1.529 (5)
N5—C21	1.290 (3)	C15—H15A	0.9800
N6—C22	1.353 (3)	C16—H16A	0.9600
N6—C21	1.398 (4)	C16—H16B	0.9600
N6—H6A	0.8600	C16—H16C	0.9600
C29—C31	1.441 (5)	C13—C14	1.365 (5)
C29—H29A	0.9600	C13—C12	1.419 (5)
C29—H29B	0.9600	C14—C8	1.403 (4)
C29—H29C	0.9600	C14—H14A	0.9300
C30—C31	1.499 (5)	C8—C9	1.388 (4)
C30—H30A	0.9600	C8—C7	1.516 (5)
C30—H30B	0.9600	C9—C11	1.404 (4)
C30—H30C	0.9600	C9—C10	1.530 (4)
C31—C32	1.515 (5)	C11—C12	1.375 (4)
C31—H31A	0.9800	C11—H11A	0.9300
C32—C37	1.371 (4)	C12—H12A	0.9300
C32—C33	1.381 (5)	C7—C6	1.503 (5)
C33—C34	1.406 (5)	C7—H7A	0.9700
C33—H33A	0.9300	C7—H7B	0.9700
C34—C35	1.387 (4)	C6—C5	1.530 (4)
C34—H34A	0.9300	C6—H6B	0.9700
C35—C36	1.409 (4)	C6—H6C	0.9700
C35—C41	1.539 (4)	C5—C4	1.552 (4)
C36—C37	1.381 (4)	C5—C10	1.560 (4)
C36—C38	1.495 (4)	C5—H5B	0.9800
C37—H37A	0.9300	C10—C1	1.548 (4)
C38—C39	1.522 (4)	C10—C20	1.555 (4)
C38—H38A	0.9700	C1—C2	1.536 (4)
C38—H38B	0.9700	C1—H1B	0.9700
C39—C40	1.537 (4)	C1—H1C	0.9700
C39—H39A	0.9700	C2—C3	1.528 (4)
C39—H39B	0.9700	C2—H2B	0.9700
C40—C41	1.555 (4)	C2—H2C	0.9700
C40—C45	1.556 (4)	C3—C4	1.532 (4)
C40—H40A	0.9800	C3—H3B	0.9700
C41—C42	1.525 (4)	C3—H3C	0.9700
C41—C46	1.550 (4)	C4—C18	1.520 (4)
C42—C43	1.522 (5)	C4—C19	1.551 (4)
C42—H42A	0.9700	C20—H20A	0.9600
C42—H42B	0.9700	C20—H20B	0.9600
C43—C44	1.542 (5)	C20—H20C	0.9600
C43—H43A	0.9700	C19—H19A	0.9600
C43—H43B	0.9700	C19—H19B	0.9600
C44—C45	1.536 (5)	C19—H19C	0.9600
C44—H44A	0.9700	C22—C23	1.499 (4)
C44—H44B	0.9700	C28—C27	1.370 (5)
C45—C48	1.543 (4)	C28—C23	1.379 (4)
C45—C47	1.555 (4)	C23—C24	1.371 (4)
C46—H46A	0.9600	C27—C26	1.365 (6)
C46—H46B	0.9600	C27—H27A	0.9300
C46—H46C	0.9600	C26—C25	1.363 (6)
C47—H47A	0.9600	C26—H26A	0.9300
C47—H47B	0.9600	C25—C24	1.383 (5)
C47—H47C	0.9600	C25—H25A	0.9300
C50—C51	1.491 (4)	C24—H24A	0.9300
			
C49—S1—C48	86.61 (14)	C54—C55—C56	119.6 (4)
C21—S2—C18	86.33 (14)	C54—C55—H55A	120.2
C48—N1—N2	113.1 (2)	C56—C55—H55A	120.2
C49—N2—N1	111.4 (2)	C55—C56—C51	121.6 (3)
C49—N3—C50	124.5 (2)	C55—C56—Cl1	117.9 (3)
C49—N3—H3A	117.8	C51—C56—Cl1	120.5 (3)
C50—N3—H3A	117.8	C15—C17—H17A	109.5
C18—N4—N5	112.7 (2)	C15—C17—H17B	109.5
C21—N5—N4	111.8 (2)	H17A—C17—H17B	109.5
C22—N6—C21	124.8 (2)	C15—C17—H17C	109.5
C22—N6—H6A	117.6	H17A—C17—H17C	109.5
C21—N6—H6A	117.6	H17B—C17—H17C	109.5
C31—C29—H29A	109.5	C16—C15—C17	112.0 (4)
C31—C29—H29B	109.5	C16—C15—C13	113.4 (3)
H29A—C29—H29B	109.5	C17—C15—C13	110.5 (3)
C31—C29—H29C	109.5	C16—C15—H15A	106.8
H29A—C29—H29C	109.5	C17—C15—H15A	106.8
H29B—C29—H29C	109.5	C13—C15—H15A	106.8
C31—C30—H30A	109.5	C15—C16—H16A	109.5
C31—C30—H30B	109.5	C15—C16—H16B	109.5
H30A—C30—H30B	109.5	H16A—C16—H16B	109.5
C31—C30—H30C	109.5	C15—C16—H16C	109.5
H30A—C30—H30C	109.5	H16A—C16—H16C	109.5
H30B—C30—H30C	109.5	H16B—C16—H16C	109.5
C29—C31—C30	114.7 (3)	C14—C13—C12	117.3 (3)
C29—C31—C32	116.8 (3)	C14—C13—C15	122.4 (3)
C30—C31—C32	111.0 (3)	C12—C13—C15	120.2 (3)
C29—C31—H31A	104.2	C13—C14—C8	123.2 (3)
C30—C31—H31A	104.2	C13—C14—H14A	118.4
C32—C31—H31A	104.2	C8—C14—H14A	118.4
C37—C32—C33	115.6 (3)	C9—C8—C14	119.9 (3)
C37—C32—C31	122.4 (4)	C9—C8—C7	122.1 (3)
C33—C32—C31	121.9 (3)	C14—C8—C7	117.9 (3)
C32—C33—C34	121.8 (3)	C8—C9—C11	116.8 (3)
C32—C33—H33A	119.1	C8—C9—C10	122.2 (3)
C34—C33—H33A	119.1	C11—C9—C10	121.0 (3)
C35—C34—C33	121.6 (4)	C12—C11—C9	123.3 (3)
C35—C34—H34A	119.2	C12—C11—H11A	118.4
C33—C34—H34A	119.2	C9—C11—H11A	118.4
C34—C35—C36	116.4 (3)	C11—C12—C13	119.5 (3)
C34—C35—C41	122.8 (3)	C11—C12—H12A	120.3
C36—C35—C41	120.8 (3)	C13—C12—H12A	120.3
C37—C36—C35	120.0 (3)	C6—C7—C8	114.6 (3)
C37—C36—C38	119.2 (3)	C6—C7—H7A	108.6
C35—C36—C38	120.8 (3)	C8—C7—H7A	108.6
C32—C37—C36	124.4 (3)	C6—C7—H7B	108.6
C32—C37—H37A	117.8	C8—C7—H7B	108.6
C36—C37—H37A	117.8	H7A—C7—H7B	107.6
C36—C38—C39	116.9 (3)	C7—C6—C5	109.8 (3)
C36—C38—H38A	108.1	C7—C6—H6B	109.7
C39—C38—H38A	108.1	C5—C6—H6B	109.7
C36—C38—H38B	108.1	C7—C6—H6C	109.7
C39—C38—H38B	108.1	C5—C6—H6C	109.7
H38A—C38—H38B	107.3	H6B—C6—H6C	108.2
C38—C39—C40	113.1 (2)	C6—C5—C4	114.9 (2)
C38—C39—H39A	109.0	C6—C5—C10	109.8 (2)
C40—C39—H39A	109.0	C4—C5—C10	117.0 (2)
C38—C39—H39B	109.0	C6—C5—H5B	104.5
C40—C39—H39B	109.0	C4—C5—H5B	104.5
H39A—C39—H39B	107.8	C10—C5—H5B	104.5
C39—C40—C41	111.0 (2)	C9—C10—C1	111.0 (2)
C39—C40—C45	112.0 (2)	C9—C10—C20	106.0 (2)
C41—C40—C45	117.6 (3)	C1—C10—C20	109.5 (2)
C39—C40—H40A	105.0	C9—C10—C5	107.7 (2)
C41—C40—H40A	105.0	C1—C10—C5	107.3 (2)
C45—C40—H40A	105.0	C20—C10—C5	115.3 (2)
C42—C41—C35	111.4 (3)	C2—C1—C10	114.2 (2)
C42—C41—C46	109.3 (3)	C2—C1—H1B	108.7
C35—C41—C46	107.5 (2)	C10—C1—H1B	108.7
C42—C41—C40	108.3 (2)	C2—C1—H1C	108.7
C35—C41—C40	105.1 (2)	C10—C1—H1C	108.7
C46—C41—C40	115.2 (2)	H1B—C1—H1C	107.6
C43—C42—C41	114.6 (3)	C3—C2—C1	110.2 (2)
C43—C42—H42A	108.6	C3—C2—H2B	109.6
C41—C42—H42A	108.6	C1—C2—H2B	109.6
C43—C42—H42B	108.6	C3—C2—H2C	109.6
C41—C42—H42B	108.6	C1—C2—H2C	109.6
H42A—C42—H42B	107.6	H2B—C2—H2C	108.1
C42—C43—C44	110.0 (3)	C2—C3—C4	112.3 (3)
C42—C43—H43A	109.7	C2—C3—H3B	109.1
C44—C43—H43A	109.7	C4—C3—H3B	109.1
C42—C43—H43B	109.7	C2—C3—H3C	109.1
C44—C43—H43B	109.7	C4—C3—H3C	109.1
H43A—C43—H43B	108.2	H3B—C3—H3C	107.9
C45—C44—C43	112.0 (3)	C18—C4—C3	108.1 (3)
C45—C44—H44A	109.2	C18—C4—C19	106.6 (2)
C43—C44—H44A	109.2	C3—C4—C19	110.7 (3)
C45—C44—H44B	109.2	C18—C4—C5	108.3 (2)
C43—C44—H44B	109.2	C3—C4—C5	108.6 (2)
H44A—C44—H44B	107.9	C19—C4—C5	114.4 (3)
C44—C45—C48	105.1 (2)	C10—C20—H20A	109.5
C44—C45—C47	110.2 (3)	C10—C20—H20B	109.5
C48—C45—C47	107.7 (2)	H20A—C20—H20B	109.5
C44—C45—C40	110.4 (3)	C10—C20—H20C	109.5
C48—C45—C40	108.4 (2)	H20A—C20—H20C	109.5
C47—C45—C40	114.7 (2)	H20B—C20—H20C	109.5
C41—C46—H46A	109.5	C4—C19—H19A	109.5
C41—C46—H46B	109.5	C4—C19—H19B	109.5
H46A—C46—H46B	109.5	H19A—C19—H19B	109.5
C41—C46—H46C	109.5	C4—C19—H19C	109.5
H46A—C46—H46C	109.5	H19A—C19—H19C	109.5
H46B—C46—H46C	109.5	H19B—C19—H19C	109.5
C45—C47—H47A	109.5	N4—C18—C4	123.5 (3)
C45—C47—H47B	109.5	N4—C18—S2	113.6 (2)
H47A—C47—H47B	109.5	C4—C18—S2	122.9 (2)
C45—C47—H47C	109.5	N5—C21—N6	119.7 (2)
H47A—C47—H47C	109.5	N5—C21—S2	115.6 (2)
H47B—C47—H47C	109.5	N6—C21—S2	124.7 (2)
N1—C48—C45	122.7 (3)	O2—C22—N6	121.3 (3)
N1—C48—S1	114.2 (2)	O2—C22—C23	123.5 (3)
C45—C48—S1	123.1 (2)	N6—C22—C23	115.2 (2)
N2—C49—N3	120.0 (2)	C27—C28—C23	121.8 (3)
N2—C49—S1	114.7 (2)	C27—C28—Cl2	117.7 (3)
N3—C49—S1	125.3 (2)	C23—C28—Cl2	120.4 (3)
O1—C50—N3	122.2 (3)	C24—C23—C28	117.5 (3)
O1—C50—C51	122.3 (3)	C24—C23—C22	118.0 (3)
N3—C50—C51	115.5 (3)	C28—C23—C22	124.5 (3)
C56—C51—C52	118.3 (3)	C26—C27—C28	119.5 (4)
C56—C51—C50	123.8 (3)	C26—C27—H27A	120.2
C52—C51—C50	117.9 (3)	C28—C27—H27A	120.2
C53—C52—C51	120.1 (3)	C25—C26—C27	120.2 (4)
C53—C52—H52A	119.9	C25—C26—H26A	119.9
C51—C52—H52A	119.9	C27—C26—H26A	119.9
C52—C53—C54	121.0 (4)	C26—C25—C24	119.7 (4)
C52—C53—H53A	119.5	C26—C25—H25A	120.1
C54—C53—H53A	119.5	C24—C25—H25A	120.1
C55—C54—C53	119.4 (3)	C23—C24—C25	121.2 (4)
C55—C54—H54A	120.3	C23—C24—H24A	119.4
C53—C54—H54A	120.3	C25—C24—H24A	119.4
			
C48—N1—N2—C49	−0.9 (4)	C50—C51—C56—Cl1	−1.1 (4)
C18—N4—N5—C21	0.8 (4)	C16—C15—C13—C14	−124.4 (4)
C29—C31—C32—C37	−121.4 (5)	C17—C15—C13—C14	108.8 (5)
C30—C31—C32—C37	104.6 (4)	C16—C15—C13—C12	58.1 (5)
C29—C31—C32—C33	61.5 (5)	C17—C15—C13—C12	−68.6 (5)
C30—C31—C32—C33	−72.5 (5)	C12—C13—C14—C8	1.7 (5)
C37—C32—C33—C34	−4.0 (5)	C15—C13—C14—C8	−175.8 (3)
C31—C32—C33—C34	173.3 (3)	C13—C14—C8—C9	0.6 (5)
C32—C33—C34—C35	1.0 (6)	C13—C14—C8—C7	177.3 (3)
C33—C34—C35—C36	2.4 (5)	C14—C8—C9—C11	−2.9 (4)
C33—C34—C35—C41	−176.2 (3)	C7—C8—C9—C11	−179.5 (3)
C34—C35—C36—C37	−2.6 (4)	C14—C8—C9—C10	179.8 (3)
C41—C35—C36—C37	176.0 (3)	C7—C8—C9—C10	3.2 (5)
C34—C35—C36—C38	178.2 (3)	C8—C9—C11—C12	3.2 (5)
C41—C35—C36—C38	−3.2 (4)	C10—C9—C11—C12	−179.5 (3)
C33—C32—C37—C36	3.8 (5)	C9—C11—C12—C13	−1.0 (5)
C31—C32—C37—C36	−173.5 (3)	C14—C13—C12—C11	−1.5 (5)
C35—C36—C37—C32	−0.5 (5)	C15—C13—C12—C11	176.1 (3)
C38—C36—C37—C32	178.6 (3)	C9—C8—C7—C6	−10.0 (5)
C37—C36—C38—C39	−161.0 (3)	C14—C8—C7—C6	173.4 (3)
C35—C36—C38—C39	18.2 (5)	C8—C7—C6—C5	40.4 (4)
C36—C38—C39—C40	10.0 (5)	C7—C6—C5—C4	159.8 (3)
C38—C39—C40—C41	−51.9 (4)	C7—C6—C5—C10	−65.7 (3)
C38—C39—C40—C45	174.4 (3)	C8—C9—C10—C1	−143.6 (3)
C34—C35—C41—C42	24.8 (4)	C11—C9—C10—C1	39.3 (4)
C36—C35—C41—C42	−153.7 (3)	C8—C9—C10—C20	97.6 (3)
C34—C35—C41—C46	−94.9 (4)	C11—C9—C10—C20	−79.6 (3)
C36—C35—C41—C46	86.6 (3)	C8—C9—C10—C5	−26.3 (4)
C34—C35—C41—C40	141.9 (3)	C11—C9—C10—C5	156.5 (3)
C36—C35—C41—C40	−36.6 (3)	C6—C5—C10—C9	56.7 (3)
C39—C40—C41—C42	−177.4 (3)	C4—C5—C10—C9	−169.9 (2)
C45—C40—C41—C42	−46.7 (3)	C6—C5—C10—C1	176.3 (2)
C39—C40—C41—C35	63.4 (3)	C4—C5—C10—C1	−50.3 (3)
C45—C40—C41—C35	−165.9 (2)	C6—C5—C10—C20	−61.3 (3)
C39—C40—C41—C46	−54.8 (4)	C4—C5—C10—C20	72.0 (3)
C45—C40—C41—C46	76.0 (3)	C9—C10—C1—C2	169.2 (2)
C35—C41—C42—C43	167.3 (3)	C20—C10—C1—C2	−74.2 (3)
C46—C41—C42—C43	−74.0 (4)	C5—C10—C1—C2	51.6 (3)
C40—C41—C42—C43	52.2 (4)	C10—C1—C2—C3	−57.7 (3)
C41—C42—C43—C44	−59.5 (4)	C1—C2—C3—C4	58.6 (3)
C42—C43—C44—C45	57.6 (4)	C2—C3—C4—C18	−172.2 (3)
C43—C44—C45—C48	−167.7 (3)	C2—C3—C4—C19	71.4 (3)
C43—C44—C45—C47	76.6 (3)	C2—C3—C4—C5	−54.9 (3)
C43—C44—C45—C40	−51.0 (4)	C6—C5—C4—C18	−59.1 (3)
C39—C40—C45—C44	177.6 (3)	C10—C5—C4—C18	169.8 (2)
C41—C40—C45—C44	47.3 (3)	C6—C5—C4—C3	−176.3 (3)
C39—C40—C45—C48	−67.8 (3)	C10—C5—C4—C3	52.7 (3)
C41—C40—C45—C48	161.9 (2)	C6—C5—C4—C19	59.6 (3)
C39—C40—C45—C47	52.5 (4)	C10—C5—C4—C19	−71.5 (3)
C41—C40—C45—C47	−77.8 (3)	N5—N4—C18—C4	−179.8 (3)
N2—N1—C48—C45	−176.9 (3)	N5—N4—C18—S2	−1.4 (3)
N2—N1—C48—S1	1.0 (3)	C3—C4—C18—N4	−118.4 (3)
C44—C45—C48—N1	83.0 (3)	C19—C4—C18—N4	0.6 (4)
C47—C45—C48—N1	−159.5 (3)	C5—C4—C18—N4	124.1 (3)
C40—C45—C48—N1	−35.0 (4)	C3—C4—C18—S2	63.3 (3)
C44—C45—C48—S1	−94.7 (3)	C19—C4—C18—S2	−177.7 (2)
C47—C45—C48—S1	22.8 (3)	C5—C4—C18—S2	−54.2 (3)
C40—C45—C48—S1	147.3 (2)	C21—S2—C18—N4	1.2 (3)
C49—S1—C48—N1	−0.7 (2)	C21—S2—C18—C4	179.7 (3)
C49—S1—C48—C45	177.2 (3)	N4—N5—C21—N6	−178.4 (3)
N1—N2—C49—N3	179.4 (3)	N4—N5—C21—S2	0.2 (3)
N1—N2—C49—S1	0.4 (3)	C22—N6—C21—N5	−176.2 (3)
C50—N3—C49—N2	177.7 (3)	C22—N6—C21—S2	5.3 (4)
C50—N3—C49—S1	−3.4 (4)	C18—S2—C21—N5	−0.8 (2)
C48—S1—C49—N2	0.1 (2)	C18—S2—C21—N6	177.8 (3)
C48—S1—C49—N3	−178.8 (3)	C21—N6—C22—O2	1.5 (5)
C49—N3—C50—O1	0.0 (5)	C21—N6—C22—C23	−179.6 (3)
C49—N3—C50—C51	−179.5 (3)	C27—C28—C23—C24	0.4 (5)
O1—C50—C51—C56	114.4 (4)	Cl2—C28—C23—C24	−177.1 (3)
N3—C50—C51—C56	−66.2 (4)	C27—C28—C23—C22	178.6 (3)
O1—C50—C51—C52	−64.6 (4)	Cl2—C28—C23—C22	1.0 (4)
N3—C50—C51—C52	114.9 (3)	O2—C22—C23—C24	58.0 (4)
C56—C51—C52—C53	0.7 (4)	N6—C22—C23—C24	−121.0 (3)
C50—C51—C52—C53	179.8 (3)	O2—C22—C23—C28	−120.2 (4)
C51—C52—C53—C54	−1.0 (5)	N6—C22—C23—C28	60.9 (4)
C52—C53—C54—C55	0.0 (5)	C23—C28—C27—C26	0.1 (6)
C53—C54—C55—C56	1.2 (5)	Cl2—C28—C27—C26	177.7 (3)
C54—C55—C56—C51	−1.5 (5)	C28—C27—C26—C25	0.3 (6)
C54—C55—C56—Cl1	−179.0 (2)	C27—C26—C25—C24	−1.1 (6)
C52—C51—C56—C55	0.5 (4)	C28—C23—C24—C25	−1.3 (5)
C50—C51—C56—C55	−178.4 (3)	C22—C23—C24—C25	−179.6 (3)
C52—C51—C56—Cl1	177.9 (2)	C26—C25—C24—C23	1.7 (6)

### Hydrogen-bond geometry (Å, °)

**Table:**

Cg is the centroid of the C23–C28 2-chlorophenyl ring.

**Table:**

*D*—H···*A*	*D*—H	H···*A*	*D*···*A*	*D*—H···*A*
N3—H3A···N5	0.86	2.03	2.882 (3)	172
N6—H6A···N2	0.86	2.14	2.982 (3)	166
C42—H42B···Cg^i^	0.97	2.65	3.462 (3)	141

Symmetry codes: (i) *x*, *y*, *z*−1.

## References

[bb6] Allen, F. H., Kennard, O., Watson, D. G., Brammer, L., Orpen, A. G. & Taylor, R. (1987). *J. Chem. Soc. Perkin Trans. 2*, pp. S1–19.

[bb1] Bruker (2001). *SMART* . Bruker AXS Inc., Madison, Wisconsin, USA.

[bb2] Bruker (2002). *SAINT* Bruker AXS Inc., Madison, Wisconsin, USA.

[bb3] Flack, H. D. (1983). *Acta Cryst.* A**39**, 876–881.

[bb4] Gu, W. & Wang, S. (2009). *Acta Cryst.* E**65**, o3270. 10.1107/S1600536809049575 PMC2972169 21578964

[bb5] Liu, X.-H., Shi, Y.-X., Ma, Y., Zhang, C.-Y., Dong, W.-L., Pan, L., Wang, B.-L., Li, B.-J. & Li, Z.-M. (2009). *Eur. J. Med. Chem.* **44**, 2782–2786. 10.1016/j.ejmech.2009.01.012 19246128

[bb7] Rao, X.-P., Song, Z.-Q., Jia, W.-H. & Shang, S.-B. (2007). *Acta Cryst.* E**63**, o3886.

[bb8] Sepulveda, B., Astudillo, L., Rodriguez, J. A., Yanez, T., Theoduloz, C. & Schmeda-Hirschmann, G. (2005). *Pharmacol. Res.* **52**, 429–437. 10.1016/j.phrs.2005.06.004 16125407

[bb9] Sheldrick, G. M. (1996). *SADABS* University of Göttingen, Germany.

[bb10] Sheldrick, G. M. (2008). *Acta Cryst.* A**64**, 112–122. 10.1107/S0108767307043930 18156677

[bb11] Song, Z.-Q. (2004). *Chem. Ind. For. Prod.* **24**(Suppl.), 7–11.

[bb12] Xu, X.-T., Duan, W.-G., Peng, Q.-H., Qin, L.-M., Li, G.-H. & Liu, X.-M. (2009). *Synth. Commun.* **39**, 2321–2328.

